# Understanding Low Motivation for Treatment in Primary Hyperparathyroidism: A Qualitative Study of Patients and Providers in the United States

**DOI:** 10.1002/wjs.70525

**Published:** 2026-08-10

**Authors:** John O'Connor, Alexandra Helbing, Diana Guiterrez‐Meza, Esra Alagoz, Alexander Chiu

**Affiliations:** ^1^ School of Medicine and Public Health University of Wisconsin Madison Wisconsin USA; ^2^ Division of Endocrine Surgery Department of Surgery School of Medicine and Public Health University of Wisconsin Madison Wisconsin USA; ^3^ Department of Surgery School of Medicine and Public Health University of Wisconsin Madison Wisconsin USA

**Keywords:** endocrine surgery, health intervention, parathyroidectomy, patient education, primary hyperparathyroidism, qualitative study

## Abstract

**Background:**

Two thirds of patients with primary hyperparathyroidism (PHPT) go untreated, risking progressive osteoporosis, fractures, and nephrolithiasis. Low patient motivation for surgery has been identified as a significant factor limiting surgical referral and treatment. We sought to better understand what patient and clinician‐level factors drive low patient motivation to identify targetable interventions.

**Study Design:**

Using a qualitative phenomenological framework, we conducted semi‐structured interviews with 19 primary care providers (PCPs), 14 patients who underwent parathyroidectomy, and 13 patients with a diagnosis of PHPT who declined intervention. Transcripts were independently coded by a multidisciplinary team using deductive thematic analysis mapped to the Disparities in Hyperparathyroidism framework to achieve thematic saturation.

**Results:**

Three main themes emerged: First, patients find PHPT abstract and hard to understand, resulting in poor disease comprehension and a misunderstanding of the risks and benefits of treatment. Second, PCPs describe difficulty explaining PHPT adequately in time‐pressured clinical visits, and patients have few, trusted outside resources for educational support. Third, this compounded misunderstanding of disease and treatment directly results in low motivation to pursue surgery. Finally, patients and PCPs both emphasized how improving patient education is key to overcoming treatment barriers.

**Conclusion:**

Untreated PHPT represents a major health service failure driven in part by poor disease literacy and constrained primary care encounters. Overcoming low surgical motivation requires the deployment of targeted and widely available patient education that clearly frame the systemic consequences of untreated disease and adequately informs risk.

## Graphical abstract

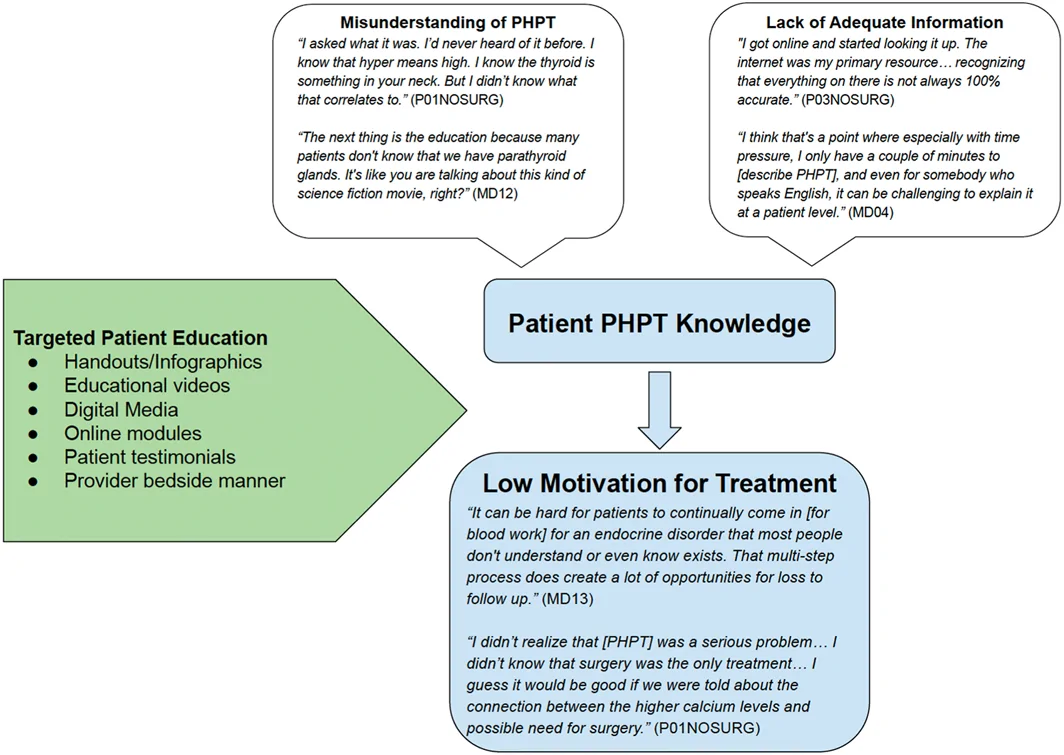

## 1 Introduction

Primary hyperparathyroidism (PHPT) is a common endocrine disorder associated with osteopenia/osteoporosis, hypertension, and nephrolithiasis [1]. The only definitive treatment for PHPT is parathyroidectomy, a safe and cost‐effective surgery with cure rates > 95% [2, 3, 4]. Despite well‐documented morbidity, PHPT remains widely underdiagnosed and undertreated, especially among socially disadvantaged patients [5, 6].

The reasons for PHPT underdiagnosis and undertreatment are multifactorial. To date, literature investigating evaluation and treatment failures has focused on system‐level barriers. Asban and colleagues reported a major contributor to low PHPT diagnosis is the lack of standardized workup following index hypercalcemia [7]. Transportation and financial difficulties have also been identified as major barriers to parathyroidectomy [8]. Access to care remains a common obstacle as approximately 1/3rd of US patients do not live within an hour drive of a specialty‐trained endocrine surgeon [9].

While structural access barriers can contribute to low treatment rates, our prior work highlighted low patient motivation for treatment can be an equally powerful barrier to care [10]. According to the COM‐B model of behavior change, promoting healthy patient behavior must address deficits in capability, opportunity, and motivation [11]. Therefore, to develop patient‐centered interventions that improve PHPT outcomes, we sought to obtain a more comprehensive understanding of what specifically contributes to low motivation for PHPT treatment. We hypothesized that patients’ poor understanding of the disease may be a modifiable factor in their motivation to seek PHPT evaluation and treatment. To investigate, we conducted qualitative interviews of primary care providers (PCPs) and two PHPT patient groups (those who underwent curative parathyroidectomy and those who declined surgery) to obtain a diverse understanding of motivational barriers to treatment and to identify potential interventions.

## 2 Materials and Methods

### 2.1 Provider Recruitment

Using purposeful sampling, we recruited PCPs (MD/DO, PA, and NP) within one Midwestern academic health network. Providers practicing in clinics serving a high proportion of patients from socially disadvantaged neighborhoods, as defined by the Area Deprivation Index (ADI), were preferentially recruited [12]. Through collaboration with our institution's Departments of Family Medicine and Internal Medicine, we distributed study information through clinic listservs.

We contacted 175 PCPs, of whom 31 were interested. Before the interviews, PCPs completed a Qualtrics (Seattle, WA) survey regarding demographic and practice information including age, race, clinic location, years of experience, and percentage of patients from disadvantaged backgrounds. Following interview completion, PCPs received a $100 gift card for their time.

### 2.2 Patient Recruitment

We established two cohorts of adult patients: those who did undergo parathyroidectomy (SURG) and those who did not undergo parathyroidectomy (NOSURG).

For the SURG group, we identified patients with a biochemically confirmed PHPT diagnosis treated with parathyroidectomy between January 2024 and May 2025. The recent surgery ensured patients could recall factors which influenced their surgical decision.

For the NOSURG group, we identified patients between January 1, 2021 and April 30, 2025 within the same health network with documented PHPT, but did not undergo parathyroidectomy. Patients were excluded if they did not receive a referral to endocrine surgery, or were diagnosed with secondary or tertiary hyperparathyroidism. We only included individuals with a surgical referral to ensure all patients were aware of and had some discussion with the PCP regarding the diagnosis and potential treatment.

We subsequently made follow‐up phone calls to ensure sample diversity regarding age, race, socioeconomic status, and sex. The preferential phone calls allowed us to better identify obstacles to treatment and management for all patients. In total, we approached 40 patients in the SURG group and 91 patients in the NOSURG group, with a total of 14 SURG group and 13 NOSURG group patient interviews completed. All patient participants received a $50 gift card for their time.

### 2.3 Data Collection

We utilized the “Disparities in the Treatment of PHPT” conceptual model to develop a semi‐structured PCP interview guide to determine their views on PHPT [13]. The provider script was pilot tested with three resident physician Zoom interviews (San Jose, CA) in June 2024. Transcripts were reviewed by all authors and interview questions were revised to receive more targeted answers about the PHPT diagnostic process and treatment barriers. The final interview script (Table S1) contained questions regarding PCP's perceptions of the PHPT diagnosis, treatment process, and potential patient resources. Our master's level qualitative researcher (DGM) completed 45–60‐min semi‐structured interviews from July to November 2024. Interviews were conducted until thematic saturation was achieved.

We created a patient interview guide (Table S2) that focused on their experiences with the PHPT diagnostic and treatment process, communication with PCPs, their decisions regarding surgical treatment, barriers to care, and potential process improvements for future patients.

### 2.4 Data Analysis

Interview recordings were de‐identified, transcribed, and imported into NVivo 14 Software (Lumivero, Denver, CO). The first four interviews for each group (PCP, SURG, and NOSURG) were coded together as a multidisciplinary analysis team (two endocrine surgeons, general surgery resident, PhD with qualitative methodology expertise, and a master's‐trained qualitative researcher). The team subsequently split into dyads to code the remainder of provider and patient transcriptions. Coders met on a weekly basis to discuss themes, resolve discrepancies, or answer ongoing questions. Lastly, we conducted NVivo queries for all codes and created data matrices to identify subthemes or representative quotes.

## 3 Results

### 3.1 Provider Demographics

Our study population included 19 PCPs from 16 different clinics. Mean cohort age was 45.0 years (SD 8). Most PCPs identified as female (68.4%), White (84.2%), and worked in an urban clinic setting (61.1%) (Table 1). Most providers (89.5%) reported seeing > 100 patients/month and 9 (47.4%) treated > 200 patients/month.

**TABLE 1:** Provider demographics (*N* = 19).

Characteristic	Value
Age in years, mean (SD)	45 (8)
Age range in years	30–60
Sex	
Male	6
Female	13
Race/ethnicity	
White	16
Asian	2
Hispanic or Latino	1
Black or African American	—
Provider type	
MD	17
PA	1
NP	1
Years in practice, mean (SD)	13 (7)
Number of patients treated per month	
0–100	2
100–150	3
150–200	5
200+	9
Provider clinic setting	
Rural	8
Urban	11

Abbreviations: MD, Doctor of Medicine; NP, Nurse Practitioner; PA, Physician Assistant; SD, Standard Deviation.

### 3.2 SURG Demographics

A total of 14 surgical patients were interviewed. Mean cohort age was 62.0 years (SD 12.8) (Table 2). The cohort predominantly identified as female (85.7%) and was composed of both White (64.2%) and Black (35.7%) participants. ADI (median 8.5, range 1–10) indicated that most participants lived in socioeconomically disadvantaged neighborhoods.

**TABLE 2:** SURG (*N* = 14) NOSURG (*N* = 13) patient demographics.

Characteristic	SURG	NOSURG
Age in years, mean (SD)	62.0 (12.8)	67.4 (18.6)
Age range in years	29–77	33–91
Sex		
Male	2	2
Female	12	11
Race/Ethnicity		
White	9	9
Black or African American	5	2
Asian	—	2
Hispanic or Latino	1	—
Insurance type		
Private	6	7
Medicaid or Medicare	8	6
Uninsured	—	—
ADI, median (range)	8.5 (1–10)	3.0 (1–10)
PHPT related comorbidities (WHO criteria)		
Age under 50	1	3
Osteopenia or osteoporosis	2	2
Nephrolithiasis	2	2
Serum calcium > 1 mg/dL above normal (10.2)	3	3
Vertebral fracture	1	1
Number of elixhauser comorbidities		
0	1	1
1	3	1
2	7	1
3	0	4
4 or more	3	6
Referral to surgery initiated by		
PCP	8	6
Endocrinology	5	5
Transplant	1	—
Urology	—	1
Rheumatology	—	1

*Note:* ADI is a validated composite measure of neighborhood socioeconomic disadvantage that analyzes 17 factors such as income, education, employment, and housing quality. Scores were assigned to patients based on their zip code. Scores range from 1 (least disadvantaged) to 10 (most disadvantaged).

Abbreviations: ADI, Area Deprivation Index; PCP, Primary Care Provider; PHPT, Primary Hyperparathyroidism; SD, standard deviation; WHO, World Health Organization.

### 3.3 NOSURG Demographics

Thirteen nonsurgical patients were interviewed. Mean cohort age was 67.4 years (SD 18.6) (Table 2). The group predominantly identified as female (84.6%) and White (69.2%). Patient insurance coverage was evenly distributed as 53.8% were privately insured and 46.2% were Medicaid/Medicare. ADI (median 3.0, range 1–10) suggested that most participants lived in socioeconomically advantaged neighborhoods.

### 3.4 Patient and Provider Interviews

**FIGURE 1:**
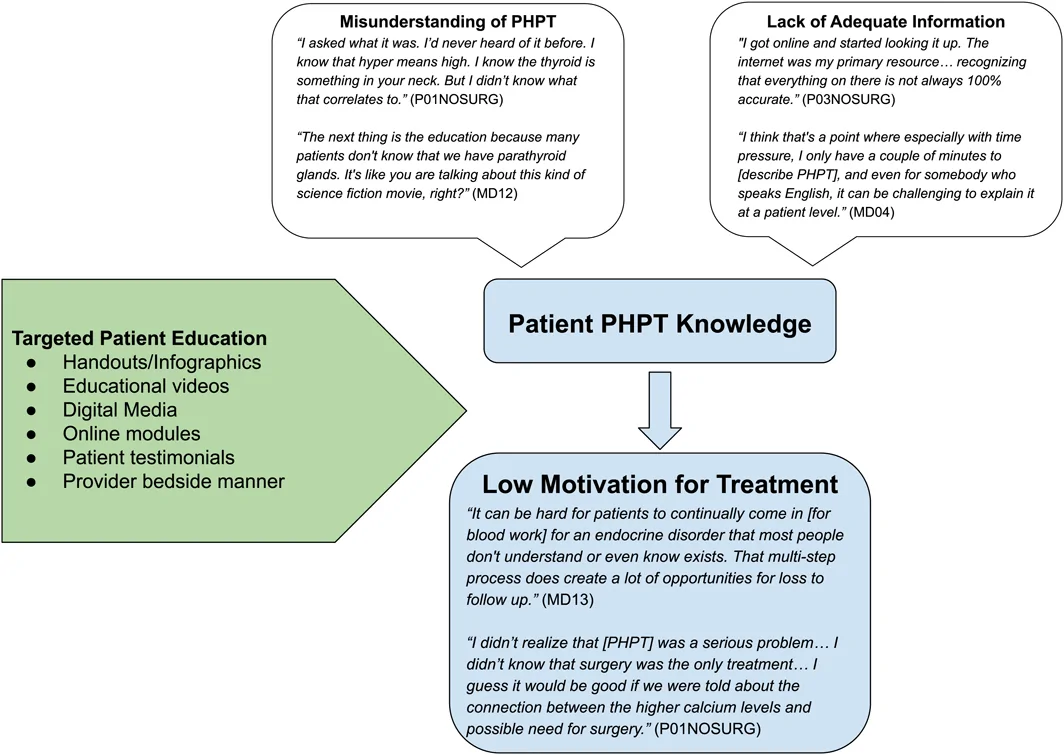
Motivational themes resulting in low PHPT treatment rates.

Qualitative interview analysis identified three sequential motivational‐related themes which impacted low PHPT treatment rates (Figure 1). First, patients commonly misunderstood the diagnosis and long‐term consequences of PHPT. Second, patients did not feel they received enough disease information to make informed decisions. Third, patients' misunderstanding directly contributed to low motivation to pursue treatment. Lastly, patients and PCPs recommended potential solutions to overcome diagnostic and treatment barriers.

### 3.5 Theme 1: Misunderstanding of PHPT

Overall, patients found PHPT confusing, and providers expressed difficulty explaining the condition clearly. One contributing factor was that most patients had no prior experience with the disease and had never heard of the parathyroid gland. As a result, patients were surprised by the diagnosis and unsure of the parathyroid's location and function.

I never even knew what a parathyroid was. I didn’t know I had one.

(P06NOSURG)

I asked what it was. I’d never heard of it before. I know that hyper means high. I know the thyroid is something in your neck. But I didn’t know what that correlates to.

(P01NOSURG)

Providers echoed how patients were often confused by the parathyroid, frequently confusing it with the thyroid. As a result, describing the physiology and pathophysiology of the parathyroid proved extremely challenging.

The next thing is the education because many patients don't know that we have parathyroid glands. It's like you are talking about this kind of science fiction movie, right?

(MD12)

I think a lot of people have heard about thyroid problems… But then when you say parathyroid, they're not really sure, and sometimes they think it's the same thing.

(MD10)

Explanation was further complicated by the fact that many consequences of PHPT lacked direct, immediately obvious physical manifestations. The vague and seemingly disconnected symptoms made it harder for patients to understand PHPT and the need for treatment.

I didn't have any symptoms, so nobody ever said anything about, you know, what it could be.

(P07SURG)

I didn’t realize that [PHPT] was a serious problem and I didn’t realize I needed to have it treated since I didn’t have any symptoms.

(P01NOSURG)

PCPs echoed this sentiment and emphasized how the lack of obvious symptoms make it difficult to explain the diagnosis and long‐term consequences.

Explaining why this could be a problem can be a challenge when patients don't necessarily feel something.

(MD04)

This is a condition that I think is a little bit harder to counsel on because it's a little bit more esoteric or not so tangible. It's less like ‘Hey, your bone is broken. You need a surgeon to fix your bone,’ or ‘You've got a growth or a tumor that we need to biopsy or take out.’

(MD19)

### 3.6 Theme 2: Lack of Adequate Information

Patients left their visits confused and wanted more information. Many patients wished providers could explain the parathyroid, PHPT, and surgery benefits more in‐depth since they left their appointment feeling uncertain about the condition.

They could’ve told me a few more things that [surgery] would’ve done if I would’ve had the parathyroid [removed]…. they didn’t really tell me.

(P06NOSURG)

No one really impressed upon me that I take this a little bit more seriously. And I don’t mean to put any blame on anybody, but, I’m a non‐medical person.

(P07_NOSURG)

PCPs similarly felt time‐limited in their ability to properly educate patients on PHPT diagnosis and treatment.

I think that's a point where especially with time pressure, I only have a couple of minutes to [describe PHPT], and even for somebody who speaks English, it can be challenging to explain it at a patient level.

(MD04)

The lack of disease understanding drove patients to look for information on their own; however, they were often not satisfied with the quality of online information.

I got online and started looking it up. The internet was my primary resource… recognizing that everything on there is not always 100% accurate.

(P03NOSURG)

I google it… I look at general information, and then I look at all the sites I can find that are provided to a layperson like myself… You know, I don't have any medical sites like physicians have.

(P10NOSURG)

### 3.7 Theme 3: Misunderstanding Leading to Low Motivation

The overall lack of disease understanding among patients culminated in low motivation to pursue the multiple required steps for diagnosis and treatment. Providers explained how they viewed low workup rate compliance as secondary to the limited understanding of the complex disease process.

It can be hard for patients to continually come in [for blood work] for an endocrine disorder that most people don't understand or even know exists. That multi‐step process does create a lot of opportunities for loss to follow up.

(MD13)

When comparing patients who underwent surgery with those who did not, a recurring theme was that understanding the consequences of untreated PHPT served as a powerful motivator for treatment. Patients who underwent curative parathyroidectomy identified more tangible symptoms and were motivated to seek treatment to prevent future health decline. Fear of worsening future symptoms and understanding the long‐term consequences reinforced their decision to proceed with surgery.

I was worried about my osteoporosis. I just thought that’s what’s sucking all the calcium out of my bones, and I realized right then, I had to get something done with it.

(P05SURG)

For many patients who did not pursue surgery, the consequences of inaction were not clear, which then manifested as a low urgency and motivation for action.

I didn’t realize that [PHPT] was a serious problem… I didn’t know that surgery was the only treatment… I guess it would be good if we were told about the connection between the higher calcium levels and possible need for surgery.

(P01NOSURG)

The [surgeon told me], “If you fall, that’s it. You go into the hospital, and then you’re done.” And I was like, “Well, how is that related to my parathyroid?” I mean, it was really weird, “make sure you don’t fall”… Maybe it’s because something happens with the parathyroid. I have no idea… I don’t know how it’s related.

(P08NOSURG)

### 3.8 Potential Solutions

The essential need for improved patient education was a common theme among providers and patients alike. Providers specifically mentioned utilizing patient education materials that were easily linked to electronic medical records.

I am a big fan of using the embedded handouts and patient instructions within HealthLink.

(MD08)

In particular, providers believed that having education specifically tailored for those with low health literacy would be useful. For example, utilizing handouts/infographics would allow patients to take information home and would assist PCPs in streamlining education during time‐limited clinic visits.

Good patient handouts would always be great. Something that really simplifies what it is, common treatments, common symptoms, reasons to come back in if they are starting to get symptoms.

(MD10)

Providers recommended the utilization of all multimedia forms (videos, radio, and podcasts) to deliver information to patients, especially for those with low literacy. This may also be helpful for patients when trying to explain the disease to family members or friends at home.

One thing that I always like to use for patients—and I just haven't found one for this particular issue—is small videos, especially for our patients who maybe can't read as well.

(MD13)

We have a radio program that we have been doing for 20 years, and we definitely bring a lot of the issues… So that is kind of the best way to communicate with the Latino community.

(MD12)

Patients shared similar views regarding the usefulness of PHPT educational materials.

Explaining where things are with a physical example like picture [would be helpful] for a lot of patients.

(P04NOSURG)

Additionally, patients who felt reassured by physicians and gained a better understanding of PHPT were more likely to proceed with treatment. Patients emphasized the importance of PCPs who took time to listen. Good bedside manner was viewed as an important aspect of the patient–provider relationship that provided comfort in moving forward with treatment.

Oh, we [PCP & I] have a very good relationship. He's always been very attentive and asks me if I have any other concerns. when I am in his office. I never, ever feel rushed.

(P01SURG)

My doctor did a good job in telling me that it's curable… and that surgery is the answer.

(P10SURG)

Finally, many patients also emphasized how testimonials or videos from other PHPT patients would prove to be an effective surgery motivator.

My friend also had the same problem. She told me she went through the surgery before me, and it’s alright. [She said,] ‘You’ve got to do it’.

(P06SURG)

## 4 Discussion

Our interviews identified several individual‐level factors contributing to poor PHPT comprehension. First, most patients were unfamiliar with the parathyroid gland and frequently confused it with the thyroid. Second, PHPT has complex and abstract pathophysiology that can be difficult to explain, especially in time‐constrained PCP visits. Lastly, the varied and nonspecific symptoms make it difficult for patients to connect their condition to the underlying diagnosis [14].

Disease misunderstanding influenced patients' decisions to pursue surgery. Compared with patients who underwent surgery, those who declined demonstrated greater misunderstanding of the disease consequences and benefits of surgical treatment. Conversely, patients who could identify specific PHPT symptoms or long‐term consequences of untreated disease were more likely to undergo surgery. The importance of disease understanding in treatment compliance has been demonstrated across multiple conditions; for example, Harsano and colleagues reported that patients with limited disease understanding were 45% less likely to undergo colorectal cancer screening compared to those with adequate disease knowledge [15]. PHPT exemplifies the necessity‐concerns framework, a theory which predicts therapy adherence based on patient belief in condition severity compared to perceived treatment risk [16]. Our findings reinforce the need to improve patient understanding of PHPT and its treatment benefits.

Both patient and provider interviews highlighted the potential value of targeted educational materials to improve disease understanding. Specifically, providers recommended developing new PHPT handouts/infographics for those with limited health literacy, as many existing resources contain excessive jargon. They emphasized items which can be reviewed at home, with family members, as clinic visit time constraints often limit comprehensive education. Patients reported spending substantial time seeking information online, expressed concerns about the trustworthiness of available resources. Their experiences are consistent with prior evaluations of online PHPT information quality [17, 18]. For example, Byrne and colleagues evaluated the top 75 search results for “parathyroidectomy” and found that most websites were of poor quality and readability [19].

The results highlight the need for reliable patient education to improve motivation for treatment. Using our findings, we plan to develop educational materials which emphasize outcomes most important to patients, while addressing common fears. Patient handouts could serve as after‐visit summaries using clear language outlining the disease process and workup, reducing the PCPs' educational burden. We also plan to develop videos describing parathyroid physiology, long term consequences of PHPT, and patient experiences with parathyroidectomy. Our multi‐faceted approach aligns with a 2024 review suggesting that the most successful interventions incorporate multiple platforms, such as digital media, educational videos, online learning modules, and mobile applications [20]. Dissemination of PHPT educational resources should be readily achievable, as providers already use similar tools for conditions such as diabetes, hypertension, and thyroid disease. The resources are also meant to be available to specialists, because even experts would benefit as it is clear that understanding and explaining the disease are two distinct entities.

Education needs can also be further tailored for providers. Our prior work demonstrated that while most PCPs felt confident managing “straightforward” PHPT patients, many felt they could use help with the work up and diagnosis of more complex cases, especially patients with vague labs or confusing presentations [21]. As a result, we created provider‐specific PHPT education as well as clearer patient referral criteria and guidelines that encourage earlier referrals.

Our study has several strengths and limitations. To our knowledge, it is the first to examine perspectives on PHPT from both patients and providers. Inclusion of multiple stakeholders allows us to better understand patient motivation and identify potential targets for intervention. A larger sample, particularly including more patients and providers from low‐resource settings and diverse geographic regions, could better inform interventions aimed at reducing disparities. Similarly, some PCPs reported minimal experience with PHPT patients. Although assessing their knowledge of PHPT guidelines and management was valuable, including providers with greater PHPT experience may have elucidated additional insight into treatment barriers. Lastly, completing interviews with other providers (e.g., endocrinologists or endocrine surgeons) may be worthwhile to explore as we seek to further increase PHPT treatment.

## 5 Conclusion

Optimizing surgical outcomes in PHPT requires improving patient understanding of the disease. Currently, vague symptomatic presentation, complex pathophysiology, and limited clinic time for patient education create substantial barriers to evidence‐based management. Future interventions should leverage educational handouts and multimodal media to improve PHPT knowledge, increase appropriate treatment, reduce disparities in care, and advance health equity.

## Author Contributions

**John O'Connor:** conceptualization, data curation, investigation, methodology, project administration, writing – original draft, writing – review and editing. **Alexandra Helbing:** conceptualization, data curation, formal analysis, funding acquisition, methodology, project administration, writing – review and editing. **Diana‐Guiterrez‐Meza:** conceptualization, data curation, formal analysis, methodology, project administration, writing – review and editing. **Esra Alagoz:** data curation, formal analysis, investigation, methodology, project administration, writing – review and editing. **Alexander Chiu:** conceptualization, data curation, formal analysis, funding acquisition, methodology, project administration, supervision, validation, writing – review and editing.

## Conflicts of Interest

The authors declare no conflicts of interest.

## Supporting information


**Table S1:** PCP interview template.

**Table S2:** Patient interview template.

## Data Availability

The data that support the findings of this study are available on request from the corresponding author. The data are not publicly available due to privacy or ethical restrictions.
