# Supplementary material for: Understanding Low Motivation for Treatment in Primary Hyperparathyroidism: A Qualitative Study of Patients and Providers in the United States
Source: World J Surg. 2026 Aug 10;50(9):2669–76. doi: 10.1002/wjs.70525 (PMC13576608; doi:10.1002/wjs.70525)
Supplement: Supplementary file 1 [file WJS-50-2669-s001.docx]

**Supplemental Table 2: Patient Interview Template**

**Diagnostic Phase:**

- **Do you have a PCP you see regularly?**
  - How long have you been under their care?
  - How often do you see them?
  - How would you describe your relationship with your PCP?
  - Do you have any issues communicating with your clinician in general?
- **Please tell me about how you were first diagnosed with primary hyperparathyroidism (PHPT).**
  - If any, what main symptoms or concerns led you to speak to your PCP?
  - What was difficult when you were getting the lab work done?
  - What helped overcome those issues?
  - At the time of your diagnosis, what was your understanding of PHPT?
  - How did you feel when you were told you had PHPT?
  - What questions or concerns did you have after hearing about your diagnosis?
- **How did your PCP or referring clinician explain the need for surgery for this condition?**
  - How did you feel when you first learned that surgery might be necessary?
  - How did your clinician communicate the urgency or importance of the surgery referral?
  - How strongly did your clinician encourage you to go see a surgeon?
  - What additional information do you wished your clinician had shared with you?
  - What resources did you use to learn more about PHPT and treatment options?

*If applicable…*

- **What language or cultural made it difficult to communicate with your clinician and understand your diagnosis or treatment options?**
- **What was your clinician’s approach to your cultural and personal values when recommending treatment options for PHPT?**

**Referral and Appointment with Surgeon:**

- **What motivated you to meet with the surgeon?**
  - What specific health concerns motivated you?
  - Did you have any reasons to not see the surgeon?
  - Looking back now, what did you wish you knew before seeing the surgeon?
- **What barriers, if any, did you encounter when setting up your appointment with your surgeon?**
  - What financial barriers did you consider during your referral to surgery? How did you find out how much insurance will cover? How did you pay for surgery?
  - What logistical challenges did you consider during your referral to surgery?
  - Were there long wait times or delays in scheduling surgery?
  - What resources helped you in the process of getting referred to surgery?
  - What resources would have made that process easier for you?

**Surgical Decision Making:**

- **What concerns or fears did you have about surgery?**
  - What did you think of the risks of having surgery?
  - What did you think of the risks of not having surgery?
  - What did you think of the benefits of having surgery?
  - Were there any specific moments or conversations that helped you feel more confident in your decision to have surgery?
  - How did your family and friends influence or support your decision to have surgery?
- **If you had to make the decision again, would you decide to have surgery for PHPT? Why?**
  - Looking back, what do you wish you would have known before having the surgery?

**Advice to Others:**

- **What advice would you give to someone else who is considering surgery for PHPT?**
- **What advice would you give to primary care clinicians talking to patients about PHPT?**
- **What advice would you give to surgeons discussing hyperparathyroidism with PHPT?**
