# Supplementary material for: Understanding Low Motivation for Treatment in Primary Hyperparathyroidism: A Qualitative Study of Patients and Providers in the United States
Source: World J Surg. 2026 Aug 10;50(9):2669–76. doi: 10.1002/wjs.70525 (PMC13576608; doi:10.1002/wjs.70525)
Supplement: Supplementary file 2 [file WJS-50-2669-s002.docx]

**Supplemental Table 1: PCP Interview Template**

**Diagnostic perception:**

- What training have you received on PHPT?
- How often do you see PHPT patients in clinic?
- How do you determine the urgency to follow up on an abnormally high calcium value?
- When specifically considering PHPT, what is your typical diagnostic workup?
- How comfortable are you with diagnosing PHPT?
- When you consider patients from disadvantaged backgrounds, what specific barriers do you as a provider encounter when trying to perform your diagnostic workup?

**Treatment perception:**

- For a patient that you made the diagnosis of PHPT, what do you do next? *[Probe on referral to surgery, endocrine, or no referral]*
- How do you decide to refer to a surgeon first versus an endocrinologist?
- What challenges do you face when referring patients to a specialist?
- Thinking of your patients from highly disadvantaged neighborhoods, what specific barriers have you as a provider encountered with getting these patients to specialist referral?

**Guidelines and resources:**

- What specific guidelines or treatment algorithms do you follow to treat PHPT?
- How can guidelines be improved to be more helpful to you?
- What resources would be helpful to explain PHPT to patients
- What other resources would you find helpful for diagnosing/referring patients? *[Probe on resources for disadvantaged communities]*
